# Risk factors for seasonal influenza virus detection in stools of patients consulting in general practice for acute respiratory infections in France, 2014‐2016

**DOI:** 10.1111/irv.12523

**Published:** 2019-05-10

**Authors:** Laëtitia Minodier, Shirley Masse, Lisandru Capai, Thierry Blanchon, Pierre‐Emmanuel Ceccaldi, Sylvie van der Werf, Thomas Hanslik, Remi Charrel, Alessandra Falchi

**Affiliations:** ^1^ EA7310, Laboratoire de Virologie Université de Corse‐Inserm Corte France; ^2^ Sorbonne Universités UPMC Univ Paris 06 INSERM, Institut Pierre Louis d’épidémiologie et de Santé Publique (IPLESP UMRS 1136) Paris France; ^3^ Pasteur Institute Virology Department Epidemiology and Physiopathology of Oncogenic Viruses Unit Paris France; ^4^ UMR CNRS 3569 Paris France; ^5^ Sorbonne Paris Cité Institut Pasteur, Cellule Pasteur Université Paris Diderot Paris France; ^6^ Pasteur Institute Virology Department Molecular Genetics of RNA Viruses Unit Paris France; ^7^ Unité de Génétique Moléculaire des Virus à ARN Université Paris Diderot Sorbonne Paris Cité Paris France; ^8^ Université Versailles Saint Quentin en Yvelines UFR de Médecine Versailles France; ^9^ Hôpital universitaire Ambroise Paré APHP Service de médecine interne Boulogne‐Billancourt France; ^10^ UMR “Emergence des Pathologies Virales” (EPV: Aix‐Marseille Univ ‐ IRD 190 ‐ Inserm 1207 ‐ EHESP) & Fondation IHU Méditerranée Infection APHM Public Hospitals of Marseille Marseille France

**Keywords:** acute respiratory infection, enteric pathogens, gastrointestinal symptoms, general practitioner, influenza virus, stool samples

## Abstract

**Background:**

Previous studies reported detection of influenza RNA in stools of patients with seasonal influenza infection. While this detection may have a clinical significance, other factors may influence the stool positivity for influenza viruses.

**Objectives:**

The objective of this study was to investigate demographical, clinical, and microbiological factors which could favor the presence of influenza viral RNA in the stools of patients with laboratory‐confirmed influenza infection.

**Methods:**

Acute respiratory infection (ARI) patients were enrolled by general practitioners (GP) during two winter seasons (2014‐2016). Nasopharyngeal swabs, stool specimens, and clinical data were collected. Samples were tested for 12 respiratory pathogen groups (nasopharyngeal and stool specimens) and for 12 enteric pathogens (stool specimens).

**Results:**

Among the 331 patients with ARI enrolled by GP, 114 (34.4%) presented influenza infection. Influenza RNA was detected in stool samples of 21% (24/114) of the 114 stool specimens analyzed. Hospitalization (adjusted odds ratio (aOR) = 7.8 (95% confidence interval (CI)) [1.7‐33.7], *P *=* *.02), age between 45 and 64 years (aOR = 4.8 [1.7‐14.5], *P *=* *.01), consumption of raw shellfish and/or mollusks (aOR = 16.7 [3.6‐90.9], *P *=* *.00), and use of antibiotics (aOR = 6.4 [2.1‐19.8], *P *=* *.006) or antiviral treatment (aOR = 7.4 [1.9‐29], *P *=* *.01) were significantly associated with an increased odds of the detection of influenza RNA in stools. Among the 24 stool samples subjected to viral isolation, no one showed virus growth.

**Conclusions:**

These findings will be useful to studies investigating the dissemination route of influenza viruses to gastrointestinal tract.

## 1 INTRODUCTION

Seasonal and pandemic influenza virus enters and replicates in cells of the upper respiratory tract where the virus recognizes sialic acid molecules linked to the Gal of glycoprotein on the surface of host epithelial cells.^1^ However, viral RNA of these viruses has been detected in stools of patients with confirmed influenza infection^2^, ^3^, ^4^, ^5^, ^6^, ^7^ with an overall reported prevalence of 20.6%.^8^ Although the main route of human influenza virus infection is respiratory, gastrointestinal (GI) symptoms such as diarrhea, nausea, vomiting, and abdominal pain are not uncommon manifestations.^9^, ^10^, ^11^, ^12^, ^13^, ^14^, ^15^ Currently, only avian influenza virus A(H5N1) is known to replicate in human intestinal tissues^16^ and to cause severe GI symptoms.^17^ While seasonal and pandemic influenza virus is likely to spread to the GI tract of patients after a primary respiratory infection, the route of dissemination remains unknown. Current knowledge explains the detection of human influenza viruses in feces because of (i) swallowing of influenza viruses from the upper respiratory tract; (ii) remnants of infected submucosal intestinal antigen‐presenting immune cells; and (iii) virus replication in intestinal cells.^8^ The significance of the detection of influenza viral RNA in stools, their dissemination on GI tract, their correlation with GI symptoms, and their viability in stool remain unclear and are still debated.^7^, ^18^

A recent study reported that while swallowed influenza seasonal viruses are vulnerable to digestive juices, artificial highly viscous mucus (imitating sputum, nasal discharge, and other mucous membrane secretions) seems to protect viral RNA and virions, allowing their detection in feces.^19^

It seems important to identify whether other factors would favor influenza detection in stools with the aim of limit interpretation biases in the future.

Thus, in this prospective study, we investigated for the first time microbiological, demographical, and clinical factors which could influence the presence of influenza viral RNA in stools of patients with laboratory‐confirmed influenza infection. Results from this study will help and inform future studies that investigate the role of human influenza virus in stools.

## 2 MATERIAL AND METHODS

### 2.1 Ethic

This study was approved by the ethics committee (CPP V, ref number 14.078). The protocol was conducted in accordance with the Helsinki Declaration. All samples were coded and tested anonymously. None of the authors collected samples. Samples were collected and sent to the test laboratory by general practitioners (GPs) involved in the research project. Patient information was stored according to national regulations, and access to such data was restricted (permission CNIL 471393). The patient's identities were not disclosed at any stage. Written informed consent was obtained from patients by the GP's. For children under the age of 18, parents or legal guardians gave permission for their participation in this project. Consent from the child was also obtained, depending on her/his age and maturity.

### 2.2 Design and study population

This is a prospective, observational, cross‐sectional study, conducted in patients consulting a GP of the French *Sentinelles* Network^20^ for an acute respiratory infection (ARI) during two winter seasons, from November 2014 to April 2015 and from November 2015 to April 2016. The case definition of ARI was “any person with a sudden onset of symptoms and at least one of the following four systemic symptoms: fever or feverishness, malaise, headache, myalgia, AND at least one of the following three respiratory symptoms: cough, sore throat, or shortness of breath.” All patients were recruited within 48 hours of the onset of symptoms.

### 2.3 Data collected

Two types of samples were obtained for each enrolled patient: a nasopharyngeal swab and a stool sample. The nasopharyngeal specimen was collected by the GP and was sent with the case report form (CRF) to the test laboratory by post in a triple packaging as required by the United Nations class 6.2 specifications. The CRF included variables such as age, sex, professional status, seasonal influenza vaccination, ARI signs and symptoms, GI symptoms, presence of chronic disease, risk factors for severe influenza, travel 15 days before consultation, drug consumption 7 days before consultation (antiviral and/or antibiotic and/or anti‐inflammatory and/or antipyretics and/or other), consumption of particular foods (tap water, oysters, mussels and/or shellfish, cooked or raw), depression, drug prescription, and required hospitalizations. Included patients were asked to collect stool specimens and send them to the laboratory within 48 hours after GP consultation by post in triple packaging as required by the United Nations class 6.2 specifications.

## 3 LABORATORY INVESTIGATIONS

### 3.1 Nucleic acid extraction

For nasopharyngeal specimens, nucleic acids were extracted from 200 μL of UTM‐stored sample and eluted in 60 μL of elution buffer using QiAamp MinElute virus spin kits (Qiagen, France) according to the manufacturer's instructions. Stool specimens were centrifuged at 20 817× *g* for 20 minutes; then, nucleic acids were extracted from 200 μL of the UTM‐stored sample and eluted in 40 μL of elution buffer using QiAamp MinElute virus spin kits (Qiagen) according to the manufacturer's instructions. An internal control (T4 and MS2 phages) was added to each extraction tube to assess the quality of the extraction at the end of the amplification.^21^

### 3.2 Quantitative reverse transcriptase polymerase chain reaction (RT‐qPCR) tests

A RT‐qPCR test was considered positive if it passed internal positive controls and had an exponential curve. Samples were excluded if the internal control value had a cycle threshold (Ct) value >35.

#### 3.2.1 Detection of influenza viruses

All extracted samples (nasopharyngeal and stool) were screened for influenza A and B viruses by RT‐qPCR; influenza A virus‐positive specimens were subtyped, and influenza B virus‐positive samples were analyzed for Victoria and Yamagata lineage according to the method developed by the French National Influenza Centre.^22^

#### 3.2.2 Detection of other respiratory pathogens

For all extracted samples (nasopharyngeal and stool), the presence of 10 non‐influenza respiratory pathogen groups was analyzed by RT‐qPCR using a Fast Track Diagnostic (FTD) Respiratory pathogens 21 kit (Fast Track Diagnostic, Luxemburg) for the presence of human *Rhinovirus* (HRV), human *Coronaviruses* NL63 (HCoV‐NL63), 229E (HCoV‐229E), OC43 (HCoV‐OC43), and HKU1 (HCoV‐HKU1), human *Parainfluenza* viruses 2, 3, and 4 (HPIV‐2, 3, and 4) and internal control, human *Parainfluenza* virus 1, *Mycoplasma pneumoniae* (*M. pneu*), human *Bocavirus* (HBoV), human *Metapneumovirus* (HMPV A/B)] and *respiratory syncytial virus* (RSVA/B), human *Adenovirus* (HAdV), *Enterovirus* (EV), and human *Parechovirus* (HPeV).

#### 3.2.3 Detection of enteric pathogens

Extracted stool samples were screened by qPCR and RT‐qPCR using the FTD viral gastroenteritis and bacterial gastroenteritis kits (Fast Track Diagnostic, Luxemburg) to detect six viruses and six bacteria: human *Norovirus* (hNoVG1 and hNoVG2), human *Adenovirus* (HAdV), human *Astrovirus* (HAstV), *Rotavirus* (RV) and *Sapovirus* (SaV), *Campylobacter coli/jejuni/lari, Escherichia coli verotoxin positive, Salmonella* spp, *Shigella* spp* + enteroinvasive Escherichia coli, Yersinia enterocolitica, Clostridium difficile*.

### 3.3 Influenza virus isolation

Nasopharyngeal and stool specimens that were positive for influenza virus were inoculated onto Madin‐Darby canine kidney (MDCK) tissue cells with an aliquot of clinical specimens and incubated for 7 days using a method reported previously.^5^

### 3.4 Statistical analysis

Quantitative variables described using median [Min‐Max] and mean with standard deviations (SD) were compared by the Wilcoxon test. Qualitative variables were described using proportions and compared using a chi‐square or Fisher's exact test if the chi‐square test was not applicable. Factors associated with the detection of influenza seasonal viruses in stools were evaluated by bivariate and multivariate logistic regression analyses. Variables for the model were chosen through automatic backwards selection using Akaike information criterion (AIC). A *P*‐value <.05 was considered statistically significant in all tests. Results were presented as odds ratio with 95% confidence intervals (OR [95% CI]). All analyses were performed using the R 3.4.1 program (http://www.r-project.org).

## 4 RESULTS

Overall, 331 ARI patients (with nasopharyngeal and stool samples) were enrolled by GPs. Influenza virus was identified in 34.4% (114/331) of nasopharyngeal samples. The median age of patients was 34 years [1‐88] and 46.5% (53/114) were male. At least one GI symptom was declared in 57.9% (66/114) of influenza cases: Diarrhea was reported by 13.2% (15/114), vomiting by 8.8% (10/114), nausea by 28.9% (33/114), and abdominal pain by 40.4% (46/114). Influenza viral RNA was detected in 21% (24 of 114) of stool specimens. Table 1 shows and compares the demographical and clinical data, and the management of influenza patients with positive (N = 24) and negative (N = 90) fecal viral RNA detection.

**Table 1:** Descriptive statistics, odds ratios, and 95% confidence intervals bivariate and multivariate models of the risks of fecal viral RNA‐positive detection

Patient Characteristics	Fecal viral RNA positiveN = 24 (%)	Fecal viral RNA negativeN = 90 (%)	OR^b^ [95% IC](*P*‐value)	aOR^c^ [95% CI](*P*‐value)
Clinical characteristics				
Male sex, no (%)	14 (58.3)	39 (43.3)	2.5 [0.8‐8.7] (0.1)	2.5 [1.0‐6.7] (0.05)
Mean age, y (SD)^a^	37.4 ± 21.4	33.3 ± 22.2		
0‐4 y	2 (8.3)	5 (5.6)	0.33 [0.01‐7.3] (0.6)	
5‐14 y	6 (25)	20 (22.2)	0.45 [0.04‐5.5] (0.6)	
15‐44 y	4 (16.6)	37 (41.1)	0.17 [0.02‐1.8] (0.2)	
45‐64 y	9 (37.5)	21 (23.3)	1.8 [0.3‐17.2] (0.6)	4.8 [1.7‐14.5] (0.01)
>65 y	3 (12.5)	7 (7.7)	0.6 [0.04‐8.4] (0.7)	
Digestive disorders <7 days	4 (16.67)	9 (10)	2.6 [0.48‐12.7] (0.3)	
Vaccination against seasonal influenza	2 (8.3)	7 (7.8)	0.4 [0.04‐46] (0.6)	
Influenza risk factors	7 (29)	27 (30)	0.6 [0.1‐2.7] (0.6)	
Hospitalization	3 (12.5)	6 (6.7)	7.0 [1.1‐45] (0.07)	7.8 [1.7‐33.7] (0.02)
High fever (>39°C)	17 (70.8)	54 (60)	2.8 [0.8‐11.7] (0.19)	2.6 [1.7‐33.7] (0.1)
Dyspnea	7 (29.2)	18 (20)	2.2 [0.6‐8.8] (0.33)	4.4 [1.1‐20] (0.05)
Gastrointestinal symptoms	13 (54.17)	53 (58.89)	0.8 [0.2‐2.7] (0.7)	
Diarrhea	4 (16.67)	11 (12.22)	1.9 [0.3‐9.5] (0.5)	
Vomiting	2 (8.33)	8 (8.89)	0.9 [0.1‐10.2] (0.9)	
Nausea	6 (25)	27 (30)	0.8 [0.2‐2.7] (0.7)	
Abdominal pain	8 (33.33)	38 (42.22)	0.6 [0.1‐3.2] (0.6)	
Food consumption				
Raw shellfish and mollusks	4 (16.67)	3 (3.33)	19.6 [2.2‐254] (0.03)	16.7 [3.6‐90.9] (0.00)
Cooked shellfish and mollusks	1 (4.17)	2 (2.22)	1.7 [0.1‐42.7] (0.7)	
Tap water	13 (54.17)	55 (61.11)	0.6 [0.1‐1.9] (0.5)	
Drug consumption before consultation				
Antibiotics	1 (4.17)	3 (3.33)	2.3 [0.1‐28] (0.5)	
Antiviral	1 (4.17)	2 (2.22)	11.7 [2.6‐56.8] (0.007)	
Drug consumption after consultation				
Antibiotics	7 (29.17)	8 (8.89)	5.2 [1.4‐22] (0.04)	6.4 [2.1‐19.8] (0.006)
Antiviral	4 (16.67)	6 (6.67)	2.7 [0.5‐12.9] (0.07)	7.4 [1.9‐29] (0.01)

a *P* = .3

b Crude odds ratios (ORs) from bivariate models.

c aOR = adjusted odds ratios from multivariate models.

CI: confidence interval.

### 4.1 Microbiological analyses of nasopharyngeal and stool samples of laboratory‐confirmed influenza patients

The detailed results from the microbiological investigation are presented in Table 2. Of the 114 nasopharyngeal samples positive for seasonal influenza viruses, 36.8% (42/114) were influenza A and 63% (72/114) were influenza B. The most frequently identified influenza virus in nasopharyngeal specimens was influenza B Victoria (50%; 57/114) followed by A(H1N1)pdm09 (21%;24/114), influenza B Yamagata (13%;15/114), and A(H3N2) (12.3%;14/114) (Table 2). Single viral infection accounted for 96.5% (110/114) of nasopharyngeal specimens, whereas multiple infections were observed in 3.5% (4/114). The four coinfections were between A(H1N1)pdm09‐HCoV‐229E, B Victoria‐HCoV‐229E, B Victoria‐HCoV‐OC43, and B Yamagata‐HCoV‐HKU1. Influenza RNA was detected in 21% (24/114) of the 114 stool specimens analyzed. Influenza B Victoria was the most detected virus (7.8%; 9/114) followed by A(H1N1)pdm09 and A(H3N2) (4.3%;5/114, respectively) and influenza B Yamagata (2.6%; 3/114).

**Table 2:** Detection rate of respiratory pathogens in nasopharyngeal and stool samples among the 114 laboratory‐confirmed influenza patients

Pathogens detected	Nasopharyngeal sampleN = 114 (%)	StoolsN = 114 (%)
Influenza A	42 (36.8)^a^	12 (10.5)^b^
*A(H1N1)pdm09*	24 (21)	*5 (4.3)*
*A(H3N2)*	14 (12.3)	*5 (4.3)*
Influenza B	72 (63)	12 (10.5)
*Victoria*	*57 (50)*	*9 (7.8)*
*Yamagata*	*15 (13)*	*3 (2.6)*
Human Coronaviruses	4 (3.5)	1 (0.8)
Human Rhinovirus	0 (0)	1 (0.8)
Single infections	110 (96.5)	113 (99)
Coinfections with respiratory viruses	4 (3.5)	0 (0)
*A(H1N1)pdm09 + HCoV‐229E*	*1 (0.8)*	
*Influenza B Victoria+ HCoV‐229E*	*1 (0.8)*	
*Influenza B Victoria+* HCoV‐OC43	*1 (0.8)*	
*Influenza B Yamagata+* HCoV‐HKU1	*1 (0.8)*	
Enteric pathogens		7 (6.1)
*HAdV*		*2 (1.7)*
*Astrovirus*		*2 (1.7)*
*Sapovirus*		*3 (2.6)*
Coinfection with respiratory bacteria	0 (0)	0 (0)
Coinfection with enteric pathogens		1 (0.8)
*A(H3N2)‐HAdV*		*1 (0.8)*

a 4 not subtyped.

b 2 not subtyped.

Among the other 12 respiratory pathogens tested in stool samples, one specimen was positive for HRV. Enteric pathogens have been detected in 6.1% (7/114) of stools. One coinfection A(H3N2)/HAdV was reported in the stools (Table 2).

The mean Ct value did not differ between nasopharyngeal samples of patients with influenza RNA‐positive stools (Ct = 25.4 ± 6.4) and patients with influenza RNA‐negative stools (Ct = 25.4 ± 6.7) (*P *=* *1). On average, in a same patient, Ct of influenza virus detection in stool sample had 6.65 more cycles than Ct of influenza virus detection in nasopharynx sample. One discrepancy was found between influenza type detection in stool (influenza A not subtyped) and nasopharyngeal sample (influenza B Yamagata) (Table 3; ID patient 2). Among the 24 stool samples subjected to viral isolation, none showed virus growth.

**Table 3:** Details of patients with influenza virus's codetection in nasopharynx and stool samples

Patient ID	Laboratory reception date	Week	Age (years)	Sex	Seasonal influenza vaccination	Gastrointestinal symptoms	Chronic disease	Risk factors	Hospitalization	Influenza virus in nasopharyngeal sample	Cycle threshold of influenza virus detected in nasopharyngeal samples	Influenza virus in stools	Cycle threshold of influenza virus detected in stool samples
1	19/01/2015	4	1	Male	No	No	No	No	No	Influenza B lineage Yamagata	26	Influenza B lineage Yamagata	34
2	06/02/2015	6	57	Male	Yes	No	No	Yes	No	Influenza B lineage Yamagata	20	A NST	34
3	11/02/2015	7	55	Male	No	No	No	No	No	Influenza B lineage Yamagata	27	Influenza B lineage Yamagata	28
4	12/02/2015	7	6	Female	No	No	Yes	Yes	Yes	A(H3N2)	33	A(H3N2)	34
5	02/03/2015	10	52	Female	No	No	No	Yes	No	A(H3N2)	25	A(H3N2)	26
6	22/01/2016	3	68	Male	No	No	Yes	Yes	Yes	Influenza B lineage Victoria	24	Influenza B lineage Victoria	28
7	26/01/2016	4	50	Female	No	No	Yes	Yes	No	A(H1N1)pdm2009	28	A(H1N1)pdm2009	34
8	03/02/2016	5	41	Female	No	No	No	No	No	Influenza B lineage Victoria	25	Influenza B lineage Victoria	32
9	24/02/2016	8	33	Male	No	No	No	No	Yes	Influenza B lineage Victoria	30	Influenza B lineage Victoria	32
10	25/02/2016	8	52	Male	No	No	No	No	No	Influenza B lineage Victoria	21	Influenza B lineage Victoria	32
11	30/03/2016	13	55	Male	No	No	No	No	No	Influenza B lineage Victoria	18	Influenza B lineage Victoria	27
12	19/12/2014	51	2	Male	No	Diarrhea and abdominal pain	No	No	No	A(H3N2)	35	A(H3N2)	35
13	08/01/2015	2	80	Female	Yes	Nausea	Yes	Yes	No	Influenza B lineage Yamagata	22	Influenza B lineage Yamagata	35
15	21/01/2015	4	62	Female	No	Abdominal pain	No	No	No	A(H3N2)	35	A(H3N2)^a^	32
16	02/02/2015	6	5	Female	No	Diarrhea, nausea and abdominal pain	No	No	No	A(H3N2)	25	A(H3N2)	33
17	15/01/2016	2	10	Female	No	Nausea and abdominal pain	No	No	No	Influenza B lineage Victoria	22	Influenza B lineage Victoria	32
18	05/02/2016	5	65	Male	No	Abdominal pain	Yes	Yes	No	A(H1N1)pdm2009	21	A(H1N1)pdm2009	32
19	10/02/2016	6	9	Female	No	Diarrhea	No	No	No	Influenza B lineage Victoria	23	Influenza B lineage Victoria	34
20	15/02/2016	7	24	Male	No	Nausea and abdominal pain	No	No	No	A(H1N1)pdm2009	34	A(H1N1)pdm2009	32
21	04/03/2016	9	43	Male	No	Vomiting	No	No	No	A(H1N1)pdm2009	21	A(H1N1)pdm2009	30
22	06/03/2016	9	13	Male	No	Vomiting and diarrhea	No	No	No	A NST	20	A NST	30
23	09/03/2016	10	56	Female	No	Nausea and abdominal pain	No	No	No	Influenza B lineage Victoria	27	Influenza B lineage Victoria	35
24	12/03/2016	10	6	Male	No	Abdominal pain	No	No	No	Influenza B lineage Victoria	24	Influenza B lineage Victoria	30
25	24/03/2016	12	54	Male	No	Nausea	No	No	No	A(H1N1)pdm2009	20	A(H1N1)pdm2009	34

a Coinfection with HadV.

#### 4.1.1 Factors associated with laboratory‐confirmed detection of seasonal influenza viruses in stools

Bivariate association between the RNA detection of influenza virus in stools (yes/no) in laboratory‐confirmed influenza patients and demographical, clinical, and management factors are presented in Table 1. Patients who had consumed raw shellfish and/or mollusks or had taken antibiotics after the GP consultation were more likely to have a positive detection of influenza virus RNA in stool (OR = 19.6 [2.2‐254], *P *=* *.03; OR = 5.2 [1.4‐22], *P *=* *.04) (Table 1). Among the four patients with exclusively influenza RNA in stools which have consumed raw shellfish and/or mollusks, three declared GI symptoms.

Multivariable analyses reported that hospitalization (aOR = 7.8 [1.7‐33.7], *P *=* *.02) was significantly associated with the fecal viral RNA detection (Table 1). Three patients (12.5% of 24 fecal viral RNA positive) have been hospitalized (ID patient 4, ID patient 6, and ID patient 9, Table 3). Two of the three hospitalized patients were positive for influenza B Victoria. The third patient was positive for A(H3N2) influenza virus. They were aged between 6 and 68 years, two were male, and two had risks factors of developing influenza complications.

Age between 45 and 64 years (aOR = 4.8 [1.7‐14.5], *P *=* *.01), consumption of raw shellfish and/or mollusks (aOR = 16.7 [3.6‐90.9], *P *=* *.00), and use of antibiotics (aOR = 6.4 [2.1‐19.8], *P *=* *.006) or antiviral treatment (aOR = 7.4 [1.9‐29], *P *=* *.01) after the GP consultation were factors significantly associated with an increased odds of the detection of influenza RNA in stools (Table 1). We noted that the confidence intervals were quite large due to small sample sizes.

## 5 DISCUSSION

This study reported for the first time the detection rate of seasonal influenza viruses in stools of laboratory‐confirmed influenza patients of all ages, in general medicine, in France. Clinical characteristics, detection of influenza viruses and other respiratory and enteric pathogens from the upper respiratory tract and in stools concomitantly, management, and risk factors for influenza RNA positivity of stool specimens were investigated.

Results of the present study suggest that age, hospitalization, and food and drug consumption seem to modify odds of detection of influenza RNA in stools of laboratory‐confirmed influenza patients. However, microbiological and clinical characteristics such as influenza virus type, Ct mean values, or GI symptoms seem to not be associated with the influenza RNA positivity of stool specimens. However, we did not report successful recovery of viable influenza virus from stools, boosting the hypothesis that intestinal cells could not support replication of influenza seasonal viruses.^5^, ^23^, ^24^

Overall, the proportion of influenza viral RNA in stool specimens reported in the present study (21%) was in line with that reported in a recent meta‐analysis (20.6% (95% CI, 8.9‐35.5)).^8^ In our study, fecal viral RNA‐positive detection ranged from 8.3% in children aged less than 5 years to 37.5% in adults belonging to the 45‐ to 64‐year age group. We observed that belonging to the age group of 45‐64 years and to be hospitalized increased the odds of detection of influenza virus in stools. This is in line with a previous study reporting positive fecal viral RNA association in stools of hospitalized adults patients,^5^ with a detection rate of 32% in patients aged between 50 and 65 years.

The source of influenza viruses in feces and how the viruses pass through the gastrointestinal tract are poorly understood. Swallowing of influenza viruses from the upper respiratory tract and hematogenous dissemination to organs through infected lymphocytes in severe influenza cases are possible explanations for fecal viral RNA detection.^5^, ^25^, ^26^ The increased odds of hospitalization in patients with influenza RNA‐positive stools seems to be more in agreement with the hematogenous dissemination of influenza viruses in severe cases with high viral load.^4^, ^27^

A positive association with the consumption of raw shellfish, and/or mollusks, and/or antibiotic and/or antiviral administration was observed. These three factors are known to increase the risk of having diarrhea, vomiting, or mild stomach symptoms resulting from the disruption of the normal microflora from drug consumption^28^, ^29^ or from the presence of enteric pathogens or toxin in food, or even intolerance to certain seafoods.^30^ Similar to other studies, no association was found between GI symptoms and influenza detection.^31^, ^32^ Interestingly, among the four patients who had influenza RNA exclusively in the stool and had consumed raw shellfish, and/or mollusks, three reported having GI symptoms, but none of the enteric pathogens screened here were detected in their stools. These symptoms could be caused by other enteric pathogens or allergy,^33^ intolerance,^34^ or toxin^35^ responsible for GI symptoms and present on shellfish and mollusks. Among the patients with antibiotic or antiviral drug prescriptions, we did not collect data on GI symptom development after GP consultation, but stool samples were collected 2 days after GP consultation; therefore, patients had started antibiotic and/or antiviral treatment at time of stool collection. We could make the assumption that disruption of the gut microflora leads to an increase in swallowing and gastric flu, which helps influenza viruses reach the lower gastrointestinal tract. A recent study reported that swallowed influenza viruses become resistant to simulated gastric acid and bile/pancreatic juice and could be detected in stools if certain protective mechanisms such as viscous mucus protect it from degradation in the GI tract.^19^

Limitations of this study include the observational study design for which residual bias cannot be excluded, and the small sample size resulting in wide confidence intervals. We did not carry out patient follow‐up to collect clinical information after GP consultation. The detection of influenza virus in stool specimens may have been reduced due to inhibitory material present in the gastrointestinal tract and stool. Ct values are a semiquantitative measure of viral load; thus, it is impossible to assign specific viral loads by pathogens.

Notwithstanding its limits, this is the first prospective multicenter study, with a standardized patient screening and a centralized confirmation of microbiological data and the simultaneous detection of respiratory and enteric pathogens in stool samples, and influenza virus isolation from stools.

We reported for the first time that several factors such as age, hospitalization, and food and drug consumption could play a role in the proportion of influenza‐positive stool samples of influenza laboratory‐confirmed infection patients. The absence of viable influenza viruses in stools seems to agree with reports showing that intestinal cells could not support replication of seasonal influenza viruses. It will be important for studies developing models to identify dissemination route of influenza viruses to GI tract and to evaluate the possibility of intestinal seasonal influenza infection, to take account of these factors.
